# Items

**Published:** 1883-03

**Authors:** 


### Items.

A meeting of the stockholders of the Chicago Dental Infirmary was held in the club rooms of the Sherman House, February 5th, for the election of officers. Drs. N. S. Davis, Wm. Byford, A. Reeves Jackson, Norman Bridge, N. B. Delamater, Gorton Nichols, Marvin Smith, Eugene S. Talbot, Frank Gardiner, Truman W. Brophy, Edgar D. Swain, James A. Swasey and A. W. Harlan were elected a board of directors. Dr. J. A. Swasey was chosen president, Dr. M. E. Smith vice-president, Dr. E. S. Talbot recording secretary, Dr. T. W. Brophy corresponding secretary, Dr. E. D. Swain treasurer. An executive committee, consisting of Drs. F. Gardiner, T. W. Brophy, E. S. Talbot, A. W. Harlan, and Gorton Nichols, was appointed, to place the infirmary upon a working basis. Rooms have been secured, and the chairs filled. It is expected to have the institution in operation by March 1st, The objects of the infirmary are : to provide the best treatment for worthy poor at a cost only of the material used, and to educate medical students, who so desire, in the art and science of dentistry. It is conceded generally that the time has come when dentistry must rank equally with other specialties in medicine. The American Medical Association has already recognized the specialty. The dental colleges are gradually requiring a more thorough education, and now nearly every chair in the medical college is included in the course, plus operative and mechanical dentistry, thus requiring more of the dental student than the medical student when only the degree of D. D. S. is conferred. Students entering the infirmary must have matriculated in some medical colleges of good standing. After attending two courses of clinical instruction and found qualified, they will receive diplomas, pro viding the degree of M. D. has been conferred. Another new departure in medical teaching was inaugurated in the medical colleges last fall. Full chairs on dental pathology and surgery were added to six of the seven colleges, and instruction on dental and oral diseases was given in the regular winter course. These chairs were filled by graduates of medicine who are practicing dentistry in Chicago. Whether the interest manifested by students was owing to the novelty of the subject or a desire to acquire a knowledge of the diseases emanating from the teeth, it certainly was very encouraging to those having charge of this branch of medicine

AN Army Medical Board has been ordered to assemble at the Army Building, corner of Houston and Greene streets, New York City, New York, March 1, 1883, for the examination of such persons as may be properly invited to present themselves before it as candidates for appointment in the Medical Corps of the army, and will probably continue in session about three months.

All candidates for appointment in the Medical Corps must apply to the Secretary of War for an invitation to appear for examination. The application must be in the handwriting of the applicant, must state date and place of his birth and place and State of which he is a permanent resident, and must be accompanied by certificates based on personal acquaintance from at least two persons of repute as to citizenship, character and moral habits : testimonials as to professional standing from Professors of the Medical College at which they graduated, should also accompany the application if they can be obtained. The candidate must be between twenty-one and twenty-eight years of age (without any exceptions), and a graduate of a regular medical college, evidence of which, his diploma, must be submitted to the Board.

Further information regarding these examinations and the nature thereof, can be obtained by addressing the Surgeon General, U. S. Army, Washington, D. C.

DEATH OF DR. MUDD, sentenced to life imprisonment for harboring J. Wilkes Booth.—Dr. Samuel A. Mudd, who served a term in the Dry Tortugas for harboring John Wilkes Booth, the assassin of President Lincoln, and assisting him to escape, died January 3d, at his residence, near Bryanstown, Charles county, South Carolina. Dr. Mudd came of an old family of prominence and influence in southern Maryland. After the assassination, Booth and Harold rode to Dr. Mudd's home, and he dressed Booth's injuries. He found that Booth's right leg was fractured, the bone being broken clear through just above the ankle. The leg was much swollen, and Dr. Mudd insisted on Booth remaining at his house all day and part of the next night. Dr. Mudd was sentenced by the court to be confined for life at hard labor, and President Johnson ordered him and others to be sent to the Albany Penitentiary. He was subsequently sent to the Dry Tortugas, where, during a yellow fever epidemic, he rendered such valuable services, that, after a few years' confinement, he was pardoned by President Johnson.—*Gaillard's Medical Journal.*

CASE OF TRAUMATIC RECTO-VESICAL-FISTULA.—Dr. W. M. Bemus, of Jamestown, N. Y., reports to us briefly an interesting case of injury to the rectum and bladder. The patient, aged about 25 years, fell from about twenty feet, striking upon a sharp stub, about two inches in diameter, which penetrated the rectum for about six inches, and entered the bladder above the prostate. Urine passed freely from the anus after the withdrawal of the stick. Treatment consisted of anodynes as needed, hot fomentations and the retention in the urethra of a flexible catheter. At the date of the report, six weeks after the injury, the patient was apparently as well as ever, with no evidence of a recto-vesical fistula, and no pain in urinating or at stool.—*Colorado Med. Journal.*

TO DISGUISE THE ODOR OF IODOFORM.—We have had many queries as to how this may be best accomplished; we therefore give every reliable report on the subject. The following is from the *New York Medical Journal and Obstetrical Review*: "Having tried nearly all the devices that have been suggested for mitigating or disguising the odor of iodoform, and found them all of little or no avail, we have lately come nearer to the object by using oil of eucalyptus, according to the following formula:

℞ Pulv. iodoform................................℥ ss

Oil eucalypt.......................................ʒ ss

Vaselin...............................................℥ iv.

M. ft. ungent.

This ointment is not without odor, but the odor is not that of iodoform."—*Med. and Surg. Rep.*, Jan. 13, 1883; *Am. Jour. Pharmacy.*

At the meeting of the Chicago Medical Society Dr. E. F. Ingals presented the following case, referred to him by Dr. Mackenzie:

Simon Ladinski, Feb. 15, 1877, was hung by a band of robbers; at the same time having his throat cut, severing the trachea. He also received two other knife wounds. After two hours he regained consciousness. Two days later was removed to a hospital; was five years and a half under the care of Prof. Schotter. After the main wound healed the glottis was found so small that only a filiform bougie cruld be passed. Dilatation was practiced steadily for about two years; the patient was then taught to use Schotter's dilator himself. He now wears the tracheal tube constantly, with plug of Schotter's dilator every night. This plug is about half an inch broad by three-eighths inch thick, and one and a half inch long. In a few months it is hoped the external wound may be closed.

THE *Illustrated Quarterly of Medicine and Surgery*, edited by Drs. George Henry Fox and F. R. Sturgis, and published by E. B. Treat, No. 757 Broadway, New York, is doubtless the most magnificent medical journal published. No. 4, of Vol. 1, which is just to hand, has nine original articles, illustrated by three chromo-lithographic plates and fifteen wood-cuts. The subscription prioe is $8 per year. We don't see how it can be done at that price, but if the publishers can stand it, the reader certainly has no reason to complain.—*Medical Chronicle.*

The Chicago Homœopathic Medical College will, after this session, cease to matriculate females. This conclusion was reached by a unanimous vote of the faculty. We congratulate them on the advancement. The co-education of the sexes in the practice of medicine and surgery must be very embarrassing and must lead to poor teaching. Women desirous of studying medicine can find every facility for the purpose in the Woman's Medical College of Chicago. The Chicago Homœopathic College has forty-five candidates for graduation.

THE Illinois State Board of Health, through its indefatigable Secretary, Dr. John Rauch, has sent us three circulars entitled "Preventable Disease Circulars," No. 2, Diphtheria; No. 3, Scarlet Fever; No. 4, Typhoid Fever. The circulars, in an admirably succinct manner, detail the methods of prevention and control in these affections. They are well worth the careful study of physicians, and we congratulate the board upon their literary and scientific merit and general distribution.

THE Health Department of Chicago, gives in its January statement the following facts: Total population 1882, 560,693; total deaths January, 966. The greatest number of deaths resulted from phthisis pulmonalis, 99; convulsions infantile, 74; diphtheria, 73; pneumonia, 74; scarlet fever, 39. There were ten deaths from suicide; thirteen deaths from small-pox. There were 212 deaths less than last January. The annual rate per 1,000 was 20.67.

ALFRED STILLE has been elected President of the Philadelphia College of Physicians, and Dr. Fordyce Barker has been re-elected President of the New York Academy of Medicine. The New York Poliklinik had sixty-four students at its first session; only legally qualified practitioners are admitted as students.—*Maryland Med. Jour.*

THE HAMMOND PRIZE.—The American Neurological Association offers a prize of five hundred dollars, to be known as the "William A. Hammond Prize," and to be awarded at the meeting in June, 1884, to the author of the best essay on the "Functions of the Thalamus in Man."

The conditions under which this prize is to be awarded are as follows.

1. The price is open to competitors of all nationalities.

2. The essays are to be based upon original observations and experiments on man and the lower animals.

3. The competing essays must be written in the English, French, or German language: if in the last, the manuscript is to be in the Italian handwriting.

4. Essays are to be sent (postage prepaid) to the Secretary of the Prize Committee, Dr. E. C. Seguin, 41 West Twentieth street, New York City, on or before February 1, 1884; each essay to be marked by a distinctive device or motto, and accompanied by a sealed envelope bearing the same device or motto, and containing the author's visiting-card.

5. The successful essay will be the property of the association, which will assume the care of its publication.

6. Any intimation tending to reveal the authorship of any of the essays submitted, whether directly or indirectly conveyed to the committee or to any member thereof, shall exclude the essay from competition.

7. The award of the prize will be announced by the undersigned committee, and will be publicly declared by the President of the association at the meeting in June, 1884.

8. The amount of the prize will be given to the successful competitor in gold coin of the United States, or, if he prefer it, in the shape of a gold medal bearing a suitable device and inscription.

Signed,

F. T. MILES, M.D., Baltimore.

J. S. JEWELL, M.D., Chicago.

E. C. SEGUIN, M.D., New York.

VACCINAL MICROCOCCI.—The Lancet says that M. Straus has plainly demonstrated the presence of the special micrococcus in microscopical preparations of the vaccinal pustule from the calf. He places the excised fragments of the skin in absolute alcohol, cuts sections and stains them with methylamine violet, and then discolors them, until only the nuclei, the bacteria and micrococci remains visible. Under a strong magnifying power the latter were visible as extremely minute points tinted blue, about a thousandth part of a millimetre in diameter, and grouped in colonies.—*Ex.*

A RECEPTION TO THE PRESIDENT AND VICE-PRESIDENT OF THE AMERICAN MEDICAL ASSOCIATION.—Dr. John V. Shoemaker, the editor of the *Medical Bulletin*, lately gave a reception at his house in Philadelphia, in honor of Dr. John L. Atlee, of Lancaster, Pa., and Dr. Alexander G. Stone, of St. Paul, Minn., the former the President and the latter the Vice-President of the American Medical Association. A great number of well-known members of the profession were present, not only from Pennsylvania, but from several of the neighboring States, and the affair is said to have been very brilliant.

THE *Illustrated Medicine and Surgery*, published by E. B. Treat, New York, under the able editorial management of Dr. Geo. H. Fox and others, is one of the most valuable of our exchanges. Its illustrations are taken from cases occurring in the practice of the best known metropolitan physicians and surgeons, and are executed with an accuracy and skill which make them exceedingly useful to the physician of every grade of proficiency and experience.

We take especial pleasure in recommending the *Illustrated Medicine and Surgery* to our readers.

DR. SEGUIN'S HEALTH.—After the tragedy which left Dr. Edward C. Seguin a childless widower, he went abroad for a change of surroundings, and has been traveling since that time in various parts of Europe. "The latest letters from him," said his brother-in-law, Dr. Amidon, recently, "show that Dr. Seguin has entirely recovered his health. It is uncertain when he will return, if ever, or whether he will resume practice in this city."

CHLORINATED SODA.—A solution of this is made as follows: To a pint of distilled water add two ounces of fresh chloride of lime, shake thoroughly, then slowly add it to a saturated solution of common washing soda until it becomes thick and turbid; let it stand until thoroughly settled, when the clear liquid should be taken off with a siphon, when it is ready for use. It should be kept tightly corked in a dark place.—*Ex.*

THE CHAIR OF ANATOMY AT HARVARD.—At a recent meeting of the Board of Overseers of Harvard University, Dr. Thos. Dwight, instructor in topographical anatomy, was appointed to fill, for the remainder of the year, the chair of anatomy, made vacant by the resignation of Professor Holmes.

TIT, BUT NOT TAT.—If any one is so wicked as to poison his grocer, who brings him food every day, the severest penalty of the law would be enforced, but if your grocer poisons you by adulterating your food, ten chances to one no notice will be taken of it.—*Ex.*

The many friends of Prof. S. J. Jewell will be pleased to learn that his health has received much benefit by his sojourn at the Hot Springs, and that he expects to resume his professional labor by the 1st of April next.

LEPROSY.—Charles Derby, a recent arrival from San Francisco, has developed into a leper at the almshouse in Salem, Mass. He was botanist to Queen Emma at Honolulu for some years.

Dr. S. B. Craver will succeed Dr. E. K. Oliver as resident physician, with Dr. Dobbins as assistant, at the Chicago Hospital for Women and Children, during the coming year.

DR. M. A. PALLEN having resigned the chair of gynæcology in the New York Post-Graduate School, Dr. B. F. Dawson has been appointed to the position.

